# Supplementary material for: Assessing Motivations and Channels for Nutritional Information Verification in Spanish University Communities
Source: Int J Environ Res Public Health. 2025 Feb 28;22(3):357. doi: 10.3390/ijerph22030357 (PMC11942194; doi:10.3390/ijerph22030357)
Supplement: Supplementary file 1 [file ijerph-22-00357-s001.zip › ijerph-3455307-supplementary.pdf]

File S1: Informed Consent

Informed Consent

To continue, please first accept our survey data policy

How are we going to treat your data?

Our commitment to privacy

The CADENUSA project is highly committed to guaranteeing your fundamental right to data protection and your right to your own image. To do this, we have adopted all the appropriate measures to comply with Regulation (EU) 2016/679 of the European Parliament and of the Council of April 27, 2016, regarding the protection of natural persons concerning the processing of personal data and the free circulation of these data (RGPD) and the applicable national law, Organic Law 3/2018, of December 5, Protection of Personal Data and Guarantee of Digital Rights (LOPDGDD, in Spanish).

Who processes my data?

The CADENUSA Project is led by the University de Valencia,
which is responsible for the treatment.
University of Valencia (UVEG)
CIF Q4618001D
Rectorate building.
Avda. Blasco Ibáñez, 13
46010 Valencia.

Who can help me if I have a question about my rights?

The data protection officer.
Javier Plaza Penadés
Data Protection Officer
Ed. Rectorate
Av.Blasco Ibañez, 13
VALÈNCIA 46010
lopd@uv.es
Telephone: 34 96 162 54 31

For what purpose is my data processed?

The contact information (email in case you decide to include it at the end of the survey) will be used to maintain contact with the participants, allowing the CADENUSA Project to share future results from this research study with them. Your answers to the questions will only be used for research purposes.

On what legal basis will my data be processed?

By accepting to participate in the study after reading this legal information, you consent to establish a relationship with CADENUSA that involves processing your identification data. This legitimation is found in Article 6.1.b) of the General Data Protection Regulation.

How long will my data be kept?

The data will be kept during the period of execution and validity of the project and, in any case, destroyed 5 years after the first publication of results.

For what purpose is my data processed?

Any personal data was required in the questionnaire: email, address, or personal data.

What are my rights?

You can exercise your rights of access, rectification, cancellation, or opposition at any time, just in case you decided to share your email. The exercise of such rights is very personal and must be done by submitting a request that must include:

▪Name and surname of the interested party.
▪Photocopy of the national identity document of the interested party or equivalent official document.
▪Identification, if applicable, of the person representing it and the document accrediting such representation.
▪ Request in which the request is specified.
▪Address for notifications, date, and signature of the applicant. ▪ Documents supporting the request made, if applicable.

The request will be addressed to:

Servei d'Informàtica - Data Protection University of Valencia Avda. Blasco Ibáñez, 13 Rectorate Building 46010 Valencia lopd@uv.es

To what authority can I complain?

The data protection authority is the Spanish Data Protection Agency.

File S2. Questionnaire: CADENUSA SURVEY—The information we receive on nutrition and food security

We are looking for creative minds to improve communication around healthy eating habits!
Have you ever wondered if the information you receive about food and nutrition is trustworthy?
Are interested in reading information on nutrition and food safety?
If so, help us by answering 4 questions

This research is part of the CADENUSA project (Awareness Campaign on Misinformation and Hoaxes in Nutrition and Food Safety), which has received funding from the UNESCO Chair and the support of the UVEG Vice-Rectorate for Internationalization and Cooperation. Likewise, it is part of the ESMODA-ECO national project, financed by the Ministry of Science and Innovation within the framework of aid for R&D projects "research challenges" in the 2018

call.

1) Are you interested in nutrition and food safety? Yes/No

1.a) Select your main motivation for nutrition and food security (choose a maximum of 2 options):

· I like taking care of my diet.
· I work or study in this field.
· I follow some kind of diet or I'm thinking about following a diet
· I like to know where my food comes from and how it's made.
· I have a medical condition, I have an allergy that requires me paying attention to my diet or I have suffered food poisoning
· Other:

1.b)Through which channels do you access to information about nutrition and food? Select from the following options the one you use more frequently(max. 3)

· Instagram
· Twitter.
· Facebook.
· TikTok.
· Youtube.
· Mainstream media (printed or digital)
· Printed/digital magazines on nutrition, food, healthy lifestyle, etc.
· Other (please indicate)

2) Do you verify the information about nutrition and food? Yes/No

2.1 How do you verify the information about nutrition? For example, do you check some relevant websites? Do you tend to doubt about the nutrition information you receive...? Write your response in the following box.

3) Regarding the information you receive about healthy food, food safety and nutrition, how would you like to know whether the information is true or it is fake news? For example, "I would like to watch stories and reels on Instagram about healthy food that also include links to specialised magazines or scientific articles" or, for instance "I think it would be a good idea that people who share information say their source, specially if are influencers the ones who share the information". Write your suggestions in the text box.

Thank you for your answers!

Our research group ScienceFlows is currently working on an educational and

training manual on good practices for verifying information on nutrition, food and food safety. If you want to receive the materials once they are finished, write your email address in the box.

CONCLUSION

We are almost finished!

We would like to thank you for your participation in this questionnaire and to hear about your experience. We are curious to know if you know about our research group ScienceFlows, about the research we carry out, the projects we do and who we collaborate with. So we leave here all the information and social networks so you can follow us and share what we do.

- Web: https://scienceflows.com/
- Instagram: https://www.instagram.com/scienceflows/
- Twitter: https://twitter.com/ScienceFlows?s=20
- Facebook: https://www.facebook.com/ScienceFlows
- YouTube: https://www.youtube.com/channel/UCXbnEIFnDvMwTmFBJLBs3SQ

If you have any questions, would like more information or would like to share with the team any experience related to nutrition and food safety misinformation, food or health hoaxes, please do not hesitate to contact us via the following email: cadenusa@scienceflows.com
